# Opioid receptors and associated regulator of G protein signaling are involved in the cathartic colon of rats

**DOI:** 10.3892/etm.2015.2233

**Published:** 2015-01-29

**Authors:** JINSONG WU, BAOHUA LIU, WEIDONG TONG, ANPING ZHANG, FAN LI, JING LIN, LI WANG

**Affiliations:** 1 Department of General Surgery, Institute of Surgery Research, Daping Hospital, Third Military Medical University, Chongqing 400042, P.R. China; 2 Second Department of General Surgery, Bethune International Peace Hospital of People’s Liberty Army, Shijiazhuang, Hebei 050082, P.R. China; 3 Department of Nephrology, Bethune International Peace Hospital of People’s Liberty Army, Shijiazhuang, Hebei 050082, P.R. China

**Keywords:** opioid receptors, constipation, cathartic colon, regulator of G protein signaling 4, β-arrestin-2

## Abstract

A cathartic colon is characteristic of slow transit constipation (STC), which can result following the long-term use of irritant laxatives. In the present study, the involvement of three opioid receptor subtypes (μ, MOR; δ, DOR; and κ, KOR), regulator of G protein signaling 4 (RGS-4) and β-arrestin-2 were investigated in the cathartic colon of rats. A rat model of a cathartic colon was established by feeding the animals with phenolphthalein, while normal rats were used as a control. The mRNA and protein expression levels of the opioid receptors, RGS-4 and β-arrestin-2 were detected in the rat colon using semi-quantitative reverse transcription polymerase chain reaction and western blot analysis, respectively. The rat model of a cathartic colon was successfully established using the phenolphthalein stimulus, and was shown to result in shrunken myenteric neurons and loose muscle fibers in the intestinal wall. The mRNA and protein expression levels of the three opioid receptor subtypes, RGS-4 and β-arrestin-2 were significantly higher in the cathartic colon group when compared with the levels in the normal control group (all P<0.01). With regard to the protein expression levels, MOR protein increased 2.4 fold, DOR expression increased 1.5 fold, KOR levels increased 1.5 fold, RGS-4 protein increased 3.5 fold and β-arrestin-2 expression increased 2.0 fold. Therefore, the expression levels of opioid receptors were found to increase in the cathartic colons of the rats, indicating that opioid receptors and downstream RGS-4 and β-arrestin-2 signaling may play an important role in the pathogenesis of STC.

## Introduction

Slow transit constipation (STC) has attracted increasing attention from medical researchers due to its serious impact on the physical and mental health and quality of life of patients. A questionnaire survey in the United States revealed that ~3% of the participants suffered from constipation (1). Among these, 15–30% of the constipation cases were STC (2). Frattini *et al* (3) defined STC as a severe functional constipation that excludes pelvic floor dysfunction, lacks physiological activity of the colon and exhibits no response to medication. Knowles *et al* (4) proposed that the causes of STC include primary intestinal neuronal or smooth muscle dysfunction, degeneration of the interstitial cells of Cajal (ICC), an autoimmune response, infectious agents, exogenous neurotoxic drugs, psychological factors, intestinal absorption dysfunction and endogenous morphine peptide and opioid receptor abnormalities. The causes of STC are not isolated, but are closely associated with each other. The existing treatment methods for STC primarily include drugs, biofeedbacks and surgical treatments. Due to the complex causes, the efficacy of STC treatment requires improvement.

A cathartic colon is an important manifestation of STC. In 1943, Heilbrun (5) proposed the concept of a cathartic colon based on X-ray findings of the colons of STC patients who had chronically used laxatives. The author reported the shrinkage or disappearance of haustra and the non-transit expansion and contraction of the colon diameter, which were similar to pathological stenosis. These observations are commonly present in the right half of the colon and often involve the terminal ileum, leading to ileocecal valve opening and ileal fold disappearance. Urso *et al* (6) pathologically examined surgical specimens of cathartic colons and identified mucosal atrophy, surface punctiform ulceration and chronic inflammation-induced reactive thickening of the muscularis mucosa, as well as submucosal fat-like infiltration and fibrosis.

STC can significantly affect patient quality of life; however, only a subset of cases ultimately require surgical treatment. Thus, human specimens for study are difficult to obtain. For this reason, Zhang *et al* (7) established a rat model of a cathartic colon by simulating the pathological changes in STC patients with chronic use of irritant laxatives. Li *et al* (8) confirmed the usefulness of the rat cathartic colon model for the study of STC.

Opiates inhibit gastrointestinal motility and secretive functions through the activation of G protein-coupled opioid receptors (9). Opioid receptors can be divided into three subtypes, including μ (MOR), δ (DOR) and κ (KOR), which are all highly expressed in the enteric nervous system (ENS) (10,11). Opiates are widely administered for the treatment of moderate to severe pain and have attracted considerable attention from medical researchers for their effects on gastrointestinal functions. A previous study reported that among patients who chronically used opiates to treat non-cancerous pain, ~40% of patients experienced constipation, compared with 7.6% in the control group. In the patients who used laxatives to treat constipation, satisfactory results were achieved in only 46% of cases. (12) STC is characterized by severe gastrointestinal dysfunction and is to a certain degree similar to opiate-induced constipation. Therefore, changes in opioid receptor expression, location and/or activity in the colon may play an important role in the course of STC.

In the present study, the involvement of opioid receptors in the course of STC was investigated by quantitative analysis of the expression levels of the opioid receptors, MOR, DOR and KOR, as well as those of regulator of G protein signaling 4 (RGS-4) and β-arrestin-2, in a cathartic colon rat model. The aim of the study was to provide a foundation for further investigation into opioid receptor function and signaling in STC patients.

## Materials and methods

### Animals

In total, 20 Wister rats (age, 7–8 weeks; male, 10; female, 10; body weight, 200±20 g) were provided by the Experimental Animal Center of the Third Military Medical University (Chongqing, China). The rats were housed in individual cages with conditions of 18–28°C and 40–80% relative humidity, access to food and water *ad libitum* and a 12-h light/dark cycle. All procedures and animal experiments were approved by the Animal Ethical Committee of the Third Military Medical University.

### Cathartic colon rat model

Animals were randomly divided into normal control and cathartic colon groups (n=10 each). Ordinary soft feed was provided for the control group, while experimental feed containing phenolphthalein (analytical reagent; Fangzheng Chemistry, Tianjin, China) was provided for the cathartic colon group. The two types of feed were provided by the Experimental Animal Center of the Third Military Medical University.

The animals were administered with an initial dose of phenolphthalein at 200 mg/kg, which was increased by 200 mg/kg daily until half of the animals had loose stools. Phenolpthalein is a type of laxative, which is taken by patients with STC to loosen stools. These patients have tolerance and dependence on the laxative and taking phenolpthalein for long periods causes stools to become less loose, requiring larger doses to have the same loosening effect (13). This dose was maintained until 80% of the animals had no loose stools. Thereafter, the animals continuously received the drug until half of the animals had loose stools. This drug protocol was repeated three times. During the final protocol, drug administration was stopped one week after loose stools had disappeared in 80% of the animals. The animals were subsequently provided with ordinary soft feed prior to further tests. The dose of the drug at the onset of diarrhea was 1,200 mg/kg, and the final dose was 3,400 mg/kg (8).

After eating a normal diet for one week, the animals were fasted for 24 h and then sacrificed by cervical dislocation. Complete colonic tissue samples were collected at ~3-cm distance from the ileocecal junction. Sub-samples of colonic tissues were stored at −70°C, and the remaining sections were fixed in 10% formalin prior to further use.

### Hematoxylin and eosin (H&E) staining

Histopathological characteristics of 10% formalin-fixed colonic tissue samples were examined by H&E staining to confirm the successful establishment of the cathartic colon rat model. The tissue samples were embedded in paraffin, cut into 4-μm sections, dewaxed with xylene and then dehydrated in a series of graded ethanol. Following washing with distilled water, the sections were stained with hematoxylin for 5 min, followed by rinsing in distilled water. Next, the sections were differentiated in hydrochloric acid-ethanol for 30 sec, immersed in warm distilled water (50°C) for 5 min and stained with eosin for 2 min. Following dehydration and clarification, the sections were mounted and fixed with neutral resin prior to examination under an XDS-500D inverted microscope (Shanghai Caikang Optical Instrument Co., Ltd., Shanghai, China).

### Semi-quantitative reverse transcription polymerase chain reaction (RT-PCR)

Colonic tissue samples were ground in liquid nitrogen, and total RNA extraction was conducted with TRIzol reagent (Tiangen Biotech Co., Ltd., Beijing, China), according to the manufacturer’s instructions. The RNA purity and concentration were determined using a UV spectrophotometer (Beckman Coulter, Brea, CA, USA). Reverse transcription of the extracted RNA (2 μg) was performed using a ReverTra Ace^®^ reverse transcription kit (Toyobo Co., Ltd., Osaka, Japan), according to the manufacturer’s instructions. Primers were designed using Primer Premier 5 software (PREMIER Biosoft International, Palo Alto, CA, USA) and are shown in Table I. The 20-μl PCR system contained 2 μl cDNA, 0.5 μl forward primer, 0.5 μl reverse primer, 10 μl Taq 2X PCR Master Mix (Tiangan Biotech Co., Ltd.), containing Taq DNA polymerase, PCR buffer, Mg2+, dNTPs, PCR stabilizer and PCR reinforing agent, and 7 μl double-distilled water. The PCR conditions were as follows: Predenaturation at 94°C for 5 min, followed by 30 cycles of denaturation at 94°C for 30 sec, annealing at 57°C for 30 sec and extension at 72°C for 30 sec, with a final extension step at 72°C for 10 min. The PCR products were subjected to 1% agarose gel electrophoresis and the relative mRNA expression levels were normalized against that of β-actin using Quantity One software (Bio-Rad, Hercules, CA, USA).

### Western blot analysis

Colonic tissue samples were ground to powder in liquid nitrogen and harvested in Eppendorf tubes (50–80 mg each). An appropriate volume (100 μl/10 mg) of radioimmunoprecipitation assay protein lysis buffer (Jinmai Biotechnology, Chongqing, China) was added to each tube. The tissue samples were lysed on ice for 30 min and then centrifuged at 13,000 xg at 4°C for 15 min. The supernatants were collected to determine the protein concentration using the bicinchoninic acid method (Beyotime Institute of Biotechnology, Haimen, China). The protein samples (80 μg each) were loaded onto 10% sodium dodecyl sulfate-polyacrylamide gels for electrophoresis. Following separation, the protein products were transferred to polyvinylidene membranes (Millipore Corporation, Billerica, MA, USA) and blocked with 5% milk powder diluted in Tris-buffered saline with 0.05% Tween 20 (TBST) for 2 h. The membranes were washed and incubated sequentially with primary antibodies (1:500) targeted against MOR (sc-27072), KOR (sc-7493), DOR (sc-7492), RGS-4 (sc-6203), β-arrestin-2 (sc-13140) and β-actin (sc-47778; Santa Cruz Biotechnology, Inc., Santa Cruz, CA, USA) at 4°C overnight. Next, the membranes were washed with TBST three times for 10 min each time. Appropriate secondary antibodies (Santa Cruz Biotechnology, Inc.) were added to the membranes, and the reaction system was shaken at room temperature for 2 h. Thereafter, the membrane was washed with TBST three times for 10 min each time. Luminescence of the protein was achieved using an enhanced chemiluminescence method (Thermo Fisher Scientific, Waltham, MA, USA). Gel images obtained from the western blot analysis assays were processed using Quantity One software. Relative protein expression levels were normalized against those of β-actin.

### Statistical analysis

All statistical analyses were conducted using SPSS version 21.0 (IBM, Armonk, NY, USA). Data are presented as the mean ± standard deviation. Differences between groups were analyzed using the independent samples t-test, where P<0.05 was considered to indicate a statistically significant difference.

## Results

### Histopathological changes in the rat cathartic colon

**Figure 1:**
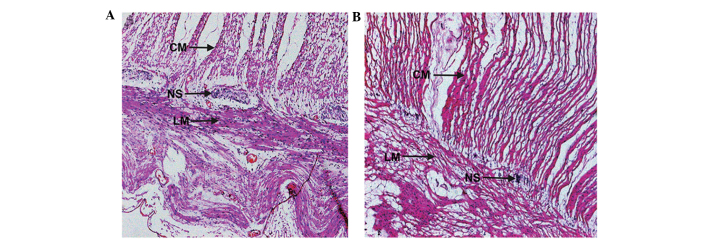
Microphotographs showing histopathological changes in rat cathartic colon revealed by hematoxylin and eosin (H&E) staining (magnification, ×500). (A) Normal colon tissue. Myenteric neurons were regular and plump, and dense muscle fibers were observed in the intestinal wall. (B) Cathartic colon tissues. Myenteric neurons were shrunken, and loose muscle fibers were present in the intestinal wall. CM: circular muscle; LM: longitudal muscle; NS: neurons.

H&E staining revealed that in the normal control rat colon, myenteric neurons were regular and plump with uniformly stained cytoplasm. In addition, dense muscle fibers were present in the intestinal wall (Fig. 1A). In the cathartic colon group, the myenteric neurons were visibly shrunken with a reduced volume, reduced cytoplasmic staining and loose muscle fibers in the intestinal wall (Fig. 1B). The histopathological changes in cathartic colon confirmed that the rat model was successfully established in all the treated animals.

### mRNA and protein expression levels of the opioid receptors in the rat cathartic colon

**Figure 2:**
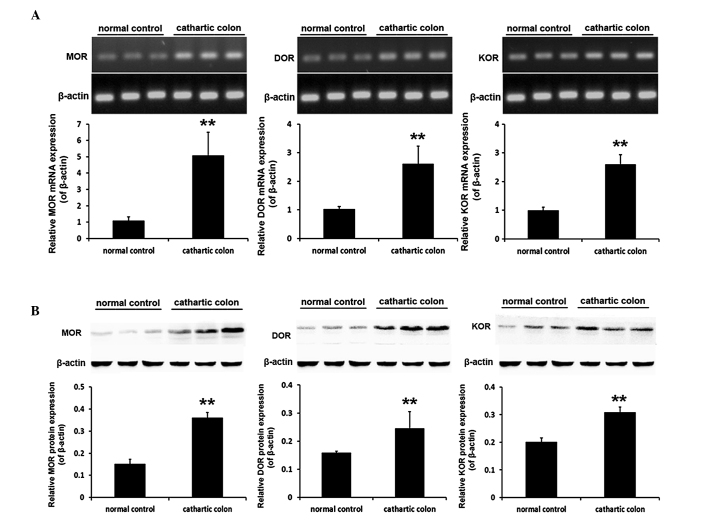
mRNA and protein expression levels of opioid receptors in the cathartic colon rat model. (A) MOR, DOR and KOR mRNA expression levels were detected using semi-quantitative reverse transcription polymerase chain reaction (n=10), where β-actin was used as an internal control. (B) MOR, DOR and KOR protein expression levels were detected by western blot analysis (n=8), where β-actin was used as an internal control. Data are expressed as the mean ± standard deviation. ^**^P<0.01, vs. control. MOR, μ-opioid receptor; DOR, δ-opioid receptor; KOR, κ-opioid receptor.

Following the successful establishment of a cathartic colon rat model using a phenolphthalein stimulus, the mRNA and protein expression levels of the three opioid receptor subtypes in the rat colon were found to be significantly higher in the cathartic colon group compared with the levels in the normal control group (all P<0.001). The mRNA expression levels of MOR were 4.7-fold greater, while DOR expression was 2.5-fold greater and KOR protein levels had increased by 2.6 fold. With regard to the protein expression levels, MOR protein was 2.4-fold greater, DOR expression had increased by 1.5 fold and KOR protein was 1.5-fold greater (Fig. 2).

### mRNA and protein expression levels of RGS-4 and β-arrestin-2 in the rat cathartic colon

**Figure 3:**
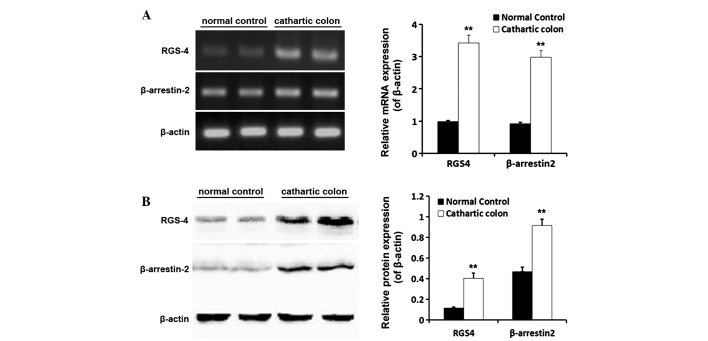
mRNA and protein expression levels of RGS-4 and β-arrestin-2 in the cathartic colon rat model. (A) RGS-4 and β-arrestin-2 mRNA expression levels were detected using semi-quantitative reverse transcription polymerase chain reaction (n=8), where β-actin was used as an internal control. (B) RGS-4 and β-arrestin-2 protein expression levels were detected by western blot analysis (n=8), where β-actin was used as an internal control. Data are expressed as the mean ± standard deviation. ^**^P<0.01, vs. control. RGS-4, regulator of G protein signaling 4.

Following successful establishment of the cathartic colon rat model using a phenolphthalein stimulus, the mRNA and protein expression levels of RGS-4 and β-arrestin-2 in the rat colon were shown to be significantly higher in the cathartic colon group when compared with those in the normal control group (all P<0.01). The mRNA expression levels of RGS-4 increased 3.4 fold, while β-arrestin-2 mRNA expression was 3.2-fold greater. With regard to the protein expression levels, RGS-4 levels were 3.5-fold greater and β-arrestin-2 expression had increased by 2.0 fold (Fig. 3).

## Discussion

The ENS is the largest and most complex nervous system outside of the central nervous system, consisting of ganglionated plexuses, the myenteric plexus between the longitudinal and circular muscles, the deep muscular plexus and the submucosal plexus, all of which are interconnected by nerve fibers to form a network system (14). The regulation of gastrointestinal physiological functions by the ENS involves multiple processes, including motility patterns, gastric acid secretion, fluid flow through epithelial cells, local blood flow changes, digestion and absorption of nutrients and interactions with the gastrointestinal immune and endocrine systems (15).

Previous studies have shown that MOR, DOR and KOR are highly expressed in the myenteric and submucosal plexuses. In addition, the receptors are distributed in nerve fibers throughout the muscle, mucosa, intestinal blood vessels, lymphatic nodes and the adjacent ICC (10,16).

In the present study, a cathartic colon rat model with typical STC was successfully established by feeding the animals with gradually increasing doses of phenolphthalein, designed to simulate the long-term use of irritant laxatives in STC patients. The results of the semi-quantitative RT-PCR and western blot analysis assays demonstrated that all three subtypes of opioid receptor (MOR, DOR and KOR) were expressed at significantly higher levels (mRNA and protein) in the cathartic colon group when compared with the control group (P<0.001). Similarly, a previous study reported that the activity of opioid receptors in the cathartic colon of rats was significantly higher compared with the control group (17) Opioid receptors are G protein-coupled metabotropic membrane receptors. Once activated, opioid receptors immediately enter cells via a concentration-dependent endocytosis mechanism, and exert biological effects through the activation of K^+^ channels, membrane hyperpolarization, Ca^2+^ channel inhibition and cyclic adenosine monophosphate generation (16). Opioid receptor agonists can simultaneously inhibit excitatory and inhibitory neurons of the ENS. Blocking the excitatory pathway can inhibit the release of excitatory neurotransmitters, including acetylcholine, thereby preventing intestinal smooth muscle tension-dependent peristaltic contractions. By contrast, blocking the inhibitory pathway can reduce the release of nitric oxide, subsequently increasing the resting tension and non-propulsive peristalsis of the smooth muscle (17). Previous studies have also demonstrated that morphine, a non-selective opioid receptor agonist, can inhibit the Na^+^ pathway in ENS neurons, which increases the action potential threshold, but reduces the amplitude, with a net effect of a lower neuronal excitability (18). Bell *et al* (19) found that 45% of patients who chronically used opioids reported less than three bowel movements per week.

In addition, opioid receptor agonists can significantly affect the movement and secretive functions of the gastrointestinal tract (20). Pol *et al* (9) reported that the upregulation of MOR expression in the intestinal tract led to intestinal dysfunction (9). Liu *et al* (21) found that MOR and KOR play an important role in regulating intestinal tract movement in a cathartic colon rat model. Furthermore, De Luca and Coupar (22) indicated that morphine and other opiates can reduce the rate of peristalsis, cause the excessive absorption of water and electrolytes from the intestinal contents and reduce intestinal fluid secretion, resulting in constipation. Wood (17) demonstrated that morphine can hyperpolarize secretomotor neurons and inhibit intestinal mucosa secretory activity. Previous studies investigating the effect of endogenous opioid peptides on the colons of STC patients revealed that met-enkephalin (23,24) and dynorphin (22) expression levels exhibited no evident abnormalities, while leu-enkephalin expression was decreased compared with the control colon (24). Ross *et al* (26) studied changes in morphine tolerance in the intestinal tract of mice and concluded that the reduced tolerance of the colon to opiates is one of the fundamental causes of gastrointestinal dysfunction. Regardless of whether the opiates are endogenous or exogenous, opioid receptors must be activated to exert their biological effects. Therefore, increased expression and enhanced activity levels of opioid receptors in the colon may play an important role in the course of STC characterized by movement disorders.

In the present study, the expression levels of RGS-4 and β-arrestin-2 were also investigated, and it was found that the expression levels were significantly higher in the cathartic colon group rats. RGS-4 is involved in the regulation of smooth muscle contraction (27), and increased levels are associated with decreased contraction (28). β-arrestins are a group of scaffolding proteins that modulate inflammatory pathways, and previous studies have indicated that β-arrestin-2 is involved in opioid-induced bowel dysfunction and opioid tolerance (29,30). The results of the present study provide evidence that these signaling pathways are likely to be involved in the pathogenesis of STC and may be potential novel therapeutic targets.

In conclusion, the experimental results demonstrated that the expression levels of MOR, DOR and KOR were significantly increased in the cathartic colons of rats. The increase in MOR expression was more evident compared with that observed for KOR and DOR expression, indicating that MOR potentially plays a more important role than the other two subtypes of opioid receptors in the course of STC. However, this hypothesis requires further investigation and more in-depth research on the three receptors. Due to the complex etiology of STC, the enhanced expression levels of opioid receptors, RGS-4 and β-arrestin-2 in the cathartic colons of the rats can be regarded as a result of the pathophysiological changes in the colon during the course of STC; however, this change cannot be considered as an initiating factor for STC based on the data presented in the current study. A large-scale epidemiological survey on STC, combined with a summary of predisposing factors and an investigation into the regulatory mechanisms of opioid receptor expression, may significantly contribute to and further the understanding into the causes of STC.

## Figures and Tables

**Table I:** RT-PCR primers.

Gene	Primer sequences (5′-3′)	Product length (bp)
β-actin		
Forward	ACCCCGTGCTGCTGACCGAG	249
Reverse	TCCCGGCCAGCCAGGTCCA	
KOR		
Forward	TCCCTGTTATCATCACCGCTGTC	210
Reverse	CTCCAAAAGGCCAAGAATTCATCA	
DOR		
Forward	CCGTTCGGAGAGCTGCTGTG	267
Reverse	GGGGAACTGGAGCGTGCATAC	
MOR		
Forward	ACCCCCCGAAATGCCAAAAT	196
Reverse	CCGGCATGATGAAAGCGAAGA	
RGS-4		
Forward	TTGGATCCATGTGCAAAGGACTCGACTAGGGAAG	198
Reverse	ATACTCGAGTTAGGCACACTGAGGGACTAGGGAAG	
β-arrestin-2		
Forward	GGGCAACTCAAGCACGAA	205
Reverse	CCTCGCAAAGTCCTCAAAC	

MOR, μ-opioid receptor; DOR, δ-opioid receptor; KOR, κ-opioid receptor; RGS-4, regulator of G protein signaling 4; RT-PCR, reverse transcription polymerase chain reaction.
